# Icariin Enhances Bone Repair in Rabbits with Bone Infection during Post-infection Treatment and Prevents Inhibition of Osteoblasts by Vancomycin

**DOI:** 10.3389/fphar.2017.00784

**Published:** 2017-10-31

**Authors:** Yang Zhang, Lifeng Shen, Zhujun Mao, Nani Wang, Xuping Wang, Xiaowen Huang, Ying Hu, Dan Shou, Chengping Wen

**Affiliations:** ^1^Department of Medicine, Zhejiang Academy of Traditional Chinese Medicine, Hangzhou, China; ^2^Department of Orthopaedic Surgery, Zhejiang Provincial Tongde Hospital, Hangzhou, China; ^3^Zhejiang Pharmaceutical College, Ningbo, China; ^4^College of Basic Medicine, Zhejiang Chinese Medical University, Hangzhou, China

**Keywords:** icariin, vancomycin, bone infection, post-infection treatment, bone repair

## Abstract

Vancomycin is an effective antibiotic for treatment of bone infection caused by *Staphylococcus aureus*, however, a high local concentration of vancomycin might induce a delay in bone union. Icariin has been reported to suppress osteoclastogenes and promote osteogenesis. Our study aimed to investigate the effect of icariin on bone repair after anti-infection treatment *in vivo* and to explore the resisting effect of icariin on rat calvarial osteoblasts (ROBs) inhibited with high doses of vancomycin. Rabbits with bone infection of *S. aureus* were treated with implanted vancomycin-calcium sulfate (VCS) and icariin at 10.86 mg/kg/day for consecutive 8 weeks. Micro-CT, morphology, blood biochemistry were evaluated. In addition, ROBs were treated with vancomycin and icariin at different doses. Cell proliferation and differentiation capabilities, BMP2, Runx2, OPG, RANKL mRNA levels and protein expression were assessed. The results indicated that high dose of vancomycin significantly decreased bone mass and inhibited osteocalcin secretion; icariin increased these indicators compared with the single vancomycin treatment. Over 0.1 mg/mL of vancomycin inhibited the proliferation and differentiation of ROBs, while icariin resisted the inhibition of vancomycin by regulating cell cycle and promoting the Alkaline phosphatase (ALP) activity. Moreover, icariin promote bone formation by up-regulating BMP2/Runx2 and OPG/RANKL pathways. Icariin exhibited osteoplastic properties on osteoblasts that had been inhibited with high doses of vancomycin. Therefore, icariin is helpful for post-infection treatment of bone infection.

## Introduction

Many factors contribute to the increase in severe bone infections and tissue suppurations, including accidents and the increased use of orthopedic devices. Bone infection has various impacts on patients, such as delayed unions and amputation (Hassani Besheli et al., 2017). Therefore, the treatment of bone infection remains a critical challenge for many orthopedic surgeons. Bone infection is mostly caused by *S. aureus*, and vancomycin is the main antibiotic that has been shown to effectively control the bone infection in the form of intravenous infusions of vancomycin and local placement of vancomycin loaded delivery vehicles (Farokhi et al., 2016; Hsu et al., 2017; Zhang et al., 2017). Nevertheless, in 10–15% of cases of bone infection, patients experienced a long bone union process, delayed union or non-union after anti-infection treatment. The factors that lead to delayed union are still unknown; some reports stated that it resulted from inflammation or even a high concentration of antibiotic medication (Eder et al., 2016; Mendoza et al., 2016). Traditionally, the main role of the local placement of VCS is to release vancomycin in the infected marrow cavity and bone to inhibit bacterium and avoid potential infection. However, the local concentrations exceeded minimal inhibitory concentrations by greater than 1000-fold in patients who received a 100 mg dose of vancomycin (Shou et al., 2014). In fact, a high dose of VCS (more than 1 g of vancomycin) was usually implanted into the local site to control the repeated infection in some cases. In addition, a previous study indicated that a high concentration of vancomycin (more than 4 mg/mL) can decrease the number of osteogenic cells, induce a loss in pseudopodia formation and alter the normal shape of the cytoplasm (Rathbone et al., 2011; Goldschmidt et al., 2016).

Chinese medicinal plants have been reported to possess osteoprotective and related properties *in vitro* and/or *in vivo* (Che et al., 2016). *Herba Epimedii* is commonly used for the treatment of fractures and other bone diseases. In our previous study, the bioactive flavonoids of *Herba Epimedii*, including icariin and epimedin A, B, and C, promoted bone formation in the rabbit model during post-infection treatment (Shou et al., 2017). Icariin is the major bioactive compound of *Herba Epimedii* (Jin et al., 2013), and has been shown to suppress osteoclastogenes in ovariectomized mice (Mok et al., 2010; Li et al., 2014), promote osteoblasts differentiation, and inhibit osteoclast differentiation (Zheng et al., 2012; Li et al., 2016).

This study was designed to evaluate the efficacy of icariin in a rabbit model of bone infection treated with VCS and to ascertain its effect on the proliferation of ROBs induced by vancomycin. In addition, the mechanism of the inhibition of vancomycin on ROBs and the reverse effect of icariin were demonstrated by RT-PCR and Western blot. The purpose of the current study was to provide a new remedy from *Herba Epimedii* to promote healing after a bone infection during the post-infection treatment with vancomycin.

## Materials and Methods

### Animal Groups and Treatment

The animal experiments were performed in accordance with the Guide for the Care and Use of Laboratory Animals and were approved by the Bioethics Committee of Zhejiang Academy of Traditional Chinese Medicine. Male New Zealand white rabbits (SCXK 2014-0047), aged 3 months and weighing 3.0 ± 0.2 kg, were housed in individual cages under air-controlled conditions (20 ± 1°C and 12 h/12 h light–dark illumination cycles), and offered the same commercial diet and tap water. The model was prepared according to previously established method (Shou et al., 2017). In total, 90% of the rabbits were diagnosed with bone infection 4 weeks after infection, and five rabbits were killed for microbiological and histological examination. As shown in **Table 1**, the rabbits in the VCS-H group, the VCS-L group and the VCS-icariin group were subjected to debridement of necrotic tissue by punching two adjacent 4-mm diameter holes in the bone, scraping and cleaning the tissue between the two holes, then implanting VCS beads (doses of vancomycin are shown in **Table 1**, rabbits in the VCS-H group were implanted 25 mg vancomycin, rabbits in the VCS-L group were implanted 12.5 mg vancomycin) in the holes during the implanting operation. In the VCS-icariin group, rabbits were implanted 25 mg vancomycin, and administered intragastrically with icariin (purity ≥ 98%, purchased from Beijing Aoke Biological Technology, Co., Ltd.) at 10.86 mg/kg/day once a day, for 8 weeks, the dose was recommended by our previous study. The control and model groups were housed under the same conditions but were not treated.

**Table 1:** Animals, groups, and treatments.

Group	Number	Model (M)	Treatment (T)		Euthanasia time point after M or T
			Vancomycin	Icariin	
Control	5	Infection	None	None	4 w after M
Model	5	Infection	None	None	4 w after M
	10	Infection	None	None	4 w and 8 w after T
VCS-H	20	Infection	25 mg	None	4 w and 8 w after T
VCS-L	20	Infection	12.5 mg	None	4 w and 8 w after T
VCS-icariin	10	Infection	25 mg	10.86 mg/kg/day	4 w and 8 w after T

### Evaluation of Anti-infection Action and Bone Formation

Specimens collected post-mortem from the bone and marrow as well as necrotic tissues collected at the time of debriding were immediately inoculated on blood agar and incubated for at least 48 h at 37°C. The identification of *S. aureus* was based on conventional criteria, including the coagulase tube test and the API Staph-Ident system (ATB 32 Staph. BioMerieux, Marcy-l’Étoile, France). The inflammatory indexes, such as CRP and WBC in serum were detected. ALP and osteocalcin in blood or tissue were measured by ELISA. ELISA kits were purchased from Beyotime Biotechnology, Co., Ltd., (Shanghai, China), and the absorbance of ALP and OC were detected by Microplate Reader (Bio-Tek, Winooski, VT, United States).

### Serum Concentrations of Icariin and Local Concentrations of Vancomycin

In the VCS-icariin group, blood was drawn from the rabbit’s auricular vein at 1, 2, 6, and 12 h after the first administration, and 2 h after the administration of icariin for 2, 3, 5, 10, 15, 20, and 30 days. The serum concentrations were determined according to the published method (Wang et al., 2015). Three rabbits in the VCS-L and VCS-H groups at the 2nd, 4th, 6th, 8th, and 15th day after treatment were executed, and the bone marrow was collected and diluted in a constant volume with normal saline. The local concentrations of vancomycin were determined via a previously established method (Shou et al., 2014).

### Micro-CT Analysis

At the 4th and 8th week, tibia specimens were examined by Micro-CT (SkyScan-1172 micro-CT scanner, Bruker, Switzerland). Oval areas 4-mm in diameter and 8-mm long were chosen as ROI (Suwanprateeb et al., 2014; Dong et al., 2017). A reconstruction of the bitmap data set was obtained and used to build the 3D model. The model was bisected with a mid-sagittal plane. Scores for the BV/TV, and BMD were obtained directly from the 3D model, following a previously study (Ren et al., 2015).

### Histological Analysis

Double labeling was performed on rabbits before they were euthanized to measure the MAR and the BFR. Calcein (20 mg/kg), alizarin red (30 mg/kg), and tetracycline (30 mg/kg) were injected intraperitoneally at 7, 14, and 21 days before euthanasia, respectively. The dissected tibia samples were fixed in 10% EDTA for 8 weeks, dehydrated in a graded series of ethanol dilutions, and then embedded in plastic sections. Unstained transverse sections (3 mm thick) were examined with a fluorescent microscope and the Osteo Measure System (OsteoMetrics, Inc.). The other tibia specimens were fixed in 4% paraformaldehyde for 24 h, decalcified in 10% EDTA for 8 weeks, dehydrated in a graded series of ethanol dilutions, and then embedded in paraffin wax. At least four consecutive 5-μm sections were obtained from the coronal planes and subjected to H&E for morphologic analysis. Transmitted light images of the stained sections were taken with a microscope (Olympus-CX41, Olympus, Japan) connected to a CCD camera (DP72; Olympus, Japan), and the images were recorded with cellSens standard software (Olympus, Japan).

### Immunohistochemical Analysis

Sections were pre-treated and stained for OC (1:100; Abcam, Ltd., Cambridge, United Kingdom) with a previously described method (Bian et al., 2012). A morphometric study was performed with an auto-analysis imaging system (Olympus-CX41, Olympus, Japan). The data were quantified with a medical image management system (Image-Pro Plus, IPP6.0).

### ROBs Culture and Treatment

Rat calvarial osteoblasts were isolated and cultured in a fully humidified incubator (37°C, 5% CO_2_) and seeded in 96-well plates at a density of 1 × 10^5^ per well. A total of 100 μL of vancomycin (0.1 mg/mL) and icariin (0.01, 0.02, 0.05 mg/mL) were added after the cells had completely adhered.

### Evaluation of Cell Proliferation and Differentiation

Cell proliferation was measured using a standard MTT assay. Prior to the end of the 24-h culture, 50 μL of MTT (5 mg/mL) was added to each well, incubated for 4 h, then the medium was discarded, and 100 μL of DMSO were added to each well. The UV absorbance was measured at 490 nm on an ELx-808 universal microplate reader (Bio-Tek) as an indicator of cell proliferation. Cell cycle analyses were evaluated by the DNA distribution and examined using propidium iodide (PI) staining (Sigma–Aldrich). ROBs were harvested after treatment vancomycin and icariin or no treatment (Control cells), fixed in 70% chilled ethanol, and kept at -20°C for at least 24 h. To measure the DNA contents, the cells were washed twice with PBS, digested with 10 mg/mL RNaseA (Sigma Aldrich) for 10 min, and then stained with 50 mg/mL PI. The DNA contents were analyzed using a flow cytometer (Becton Dickinson, Mountain View, CA, United States). ALP is an enzyme expressed by cells, and its activity is a well-defined marker for osteogenesis. ALP is a marker of bone formation and differentiation of osteoblasts. ALP activity was measured according to the literature (Jiao et al., 2009).

### Real-time PCR

Total RNA was extracted from ROBs using TRIzol (Invitrogen Life Technologies, Carlsbad, CA, United States). Reverse transcription was performed with an RT-for-PCR kit (Qiagen, Valencia, CA, United States) following the manufacturer’s protocol. Real-time PCR was performed with SYBR Premix Ex Taq (Takara Bio) in an RG3000 machine (Corbett Research, Australia). Target mRNA expression was normalized to β-actin expression. PCR products were subjected to melt curve analysis, and the data were quantified with Rotor Gene 6.0 analysis software.

### Western Blot Analysis

Proteins from ROBs were extracted with E-PER protein extraction reagents (Thermo Scientific, Waltham, MA, United States), transferred onto a PVDF membrane (Bio-Rad, Hercules, CA, United States) blocked with 5% non-fat milk in PBST at room temperature. After an overnight incubation with the primary antibody at 4°C and incubation with the HRP-conjugated secondary antibodies (Thermo Scientific, Waltham, MA, United States) at RT, protein expression was detected with a Super Signal West Femto Maximum Sensitivity Substrate Kit (Thermo Scientific, Waltham, MA, United States). The anti-GAPDH polyclonal mouse antibody (Sigma, St. Louis, MO, United States), anti-BMP2 polyclonal rabbit antibody, anti-OPG polyclonal rabbit antibody, and anti-RANKL polyclonal rabbit antibody (Abcam, Cambridge, MA, United States) served as primary antibodies.

### Statistical Analysis

The data are presented as the mean ± SD, and statistical significance was calculated via one-way analysis of variance followed by a *post hoc* least significant difference test (homogeneity of variance) or the Tukey test (heterogeneity of variance) with SPSS 20.0 software (SPSS, Inc., Chicago, IL, United States). The significance level was defined as *P* < 0.05 or *P* < 0.01.

## Results

### Analysis of Serum and Local Tissue Concentration

**FIGURE 1:**
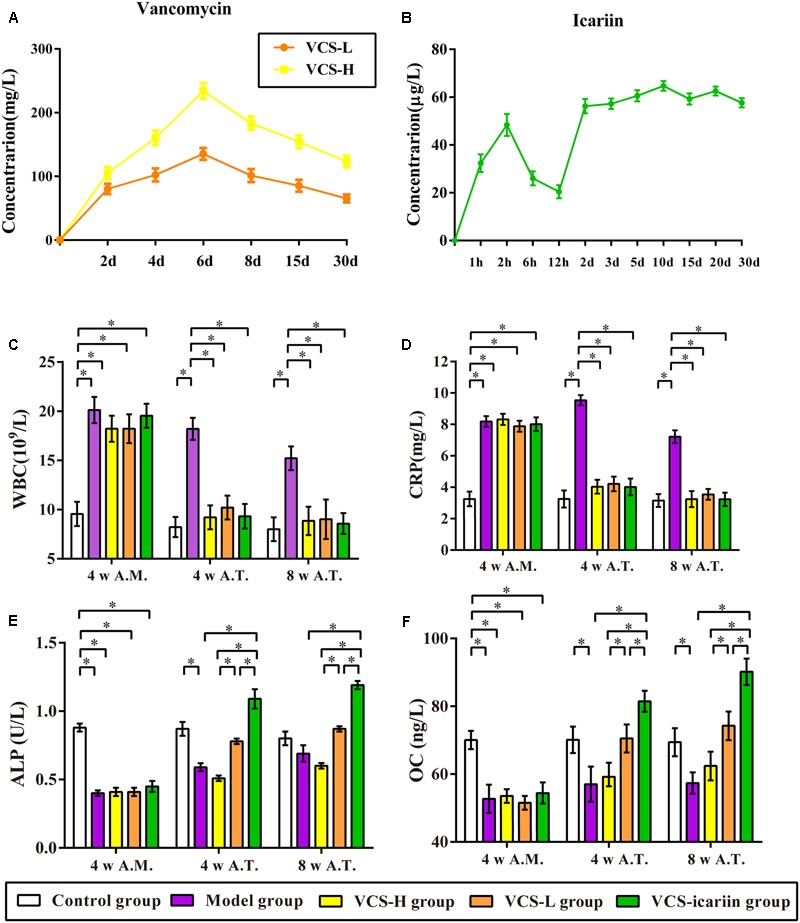
**(A)** Local concentration-time curves of vancomycin. **(B)** Serum concentration–time curve of icariin. **(C–F)** The results of CRP, WBC, ALP, and osteocalcin in the rabbit serum at the fourth week after infection and the fourth week and eighth week after treatment, ^∗^*P* < 0.05. 4 w A.M., 4 weeks after the model was established; 4 w A.T., 4 weeks after treatment; 8 w A.T., 8 weeks after treatment.

The local concentration-time curve of vancomycin is shown in **Figure 1A**. The local vancomycin concentrations were significantly higher within 6 days after the implantation of VCS-H and VCS-L, and reached peak levels at 234.21 and 135.37 mg/L, respectively. At the 30th day, the concentrations of VCS-H and VCS-L were reduced to 123.37 and 65.32 mg/L, respectively. As shown in **Figure 1B**, the serum concentration of icariin reached 48.39 μg/L 2 h after the first administration and maintained at a steady level of 50 μg/L in the following 30 days, which illustrated that icariin could be absorbed in the blood circulation and detected.

### Anti-infection and Bone Formation Assessments

The levels of WBC and CRP in the model group were more than double the levels in the control group at the 4th week after the model was established (**Figures 1C,D**). VCS-H and VCS-L treatment significantly decreased the levels of WBC from 18.21 × 10^9^/L to less than 10 × 10^9^/L, and decreased the levels of CRP from 9.54 to 4 mg/L at the 4th week after treatment. There was no significant difference in CRP or WBC in the control group, VCS-H, VCS-L, and VCS-icariin groups after treatment, which illustrated that VCS had an excellent anti-inflammatory effect. As shown in **Figures 1E,F**, ALP and OC in serum were decreased by 50% at the 4th week after the model was established, and the levels were improved by VCS-icariin after treatment. However, serum ALP and OC in the VCS-H group were significantly lower than in the VCS-L group after treatment. These results indicated that the high local concentration of vancomycin reduced the levels of bone formation indicators in the peripheral blood. On the other hand, serum ALP and OC could be increased via the intragastric administration of icariin.

### Icariin Promoted Bone Formation during the Post-infection Treatment Period

**FIGURE 2:**
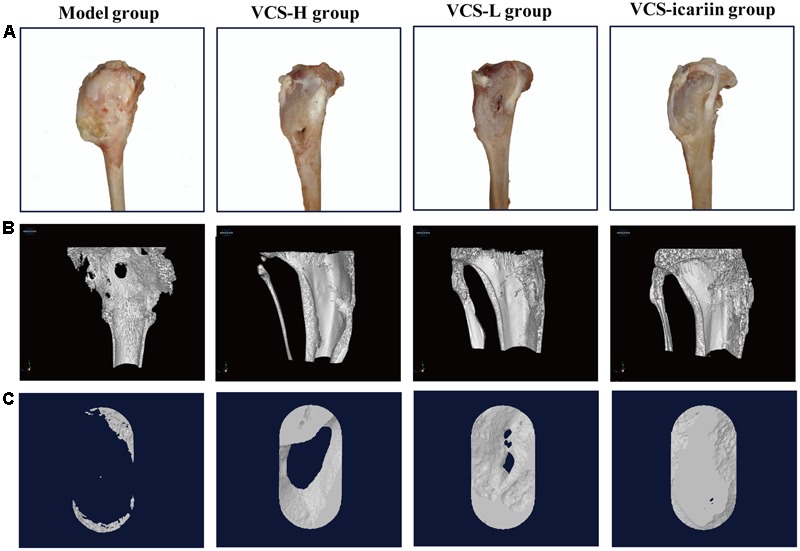
**(A)** Appearance of the rabbit tibias 8 weeks after treatment with VCS-L, VCS-H, and VCS-icariin. **(B)** Three-dimensional reconstruction of bone defects of the tibia in bone infection model rabbits that underwent 8 weeks of treatment with VCS-L, VCS-H, and VCS-icariin. **(C)** Three-dimensional reconstruction of the bone defect of the ROI in the model, VCS-L, VCS-H, and VCS-icariin groups.

At the 8th week after treatment, the rabbits in the model group had a large number of infected sinuses, and white and yellow pus overflowed from the wounds. The bone defects in the VCS-icariin group were the smallest in the three treatment groups, and those in the VCS-H group were the largest (**Figure 2A**). The bone defect in the model group was evident in the Micro-CT results of three-dimensional reconstruction figures of the tibia and the ROI (**Figures 2B,C**). The defect areas in the VCS-L, VCS-H and VCS-icariin groups were significantly reduced, moreover, the bone mass was more improved in the VCS-icariin group than in the VCS-L and VCS-H groups. As shown in **Table 2**, the indicators of BV/TV and BMD in the model group were 10.34 ± 1.62% and 0.32 ± 0.03 g/cm^2^ at the 8th week after treatment. The VCS-icariin treatment increased BV/TV and BMD to 75.33 ± 4.25% and 1.26 ± 0.04 g/cm^2^, respectively, which were significantly higher than the indicators in the VCS-H group. These results indicated that bone healing after the anti-infection treatment was influenced by a high local concentration vancomycin; however, icariin noticeably promoted bone repair.

**Table 2:** Effects of icariin on tibia microarchitecture in the rabbits (*n* = 3).

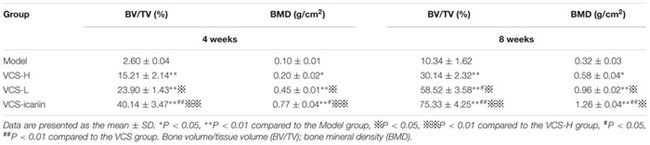

**FIGURE 3:**
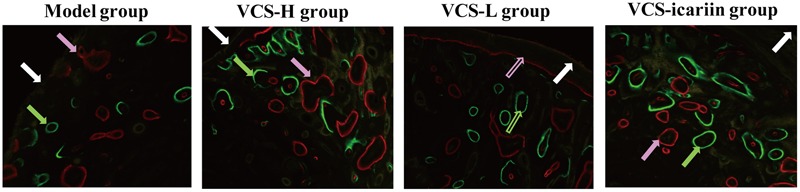
Typical fluoroscope photograph of the tibia with triple fluorescent labeling. White arrows: tetracycline labeling, pink arrows: alizarin red labeling, green arrows: calcein labeling.

**FIGURE 4:**
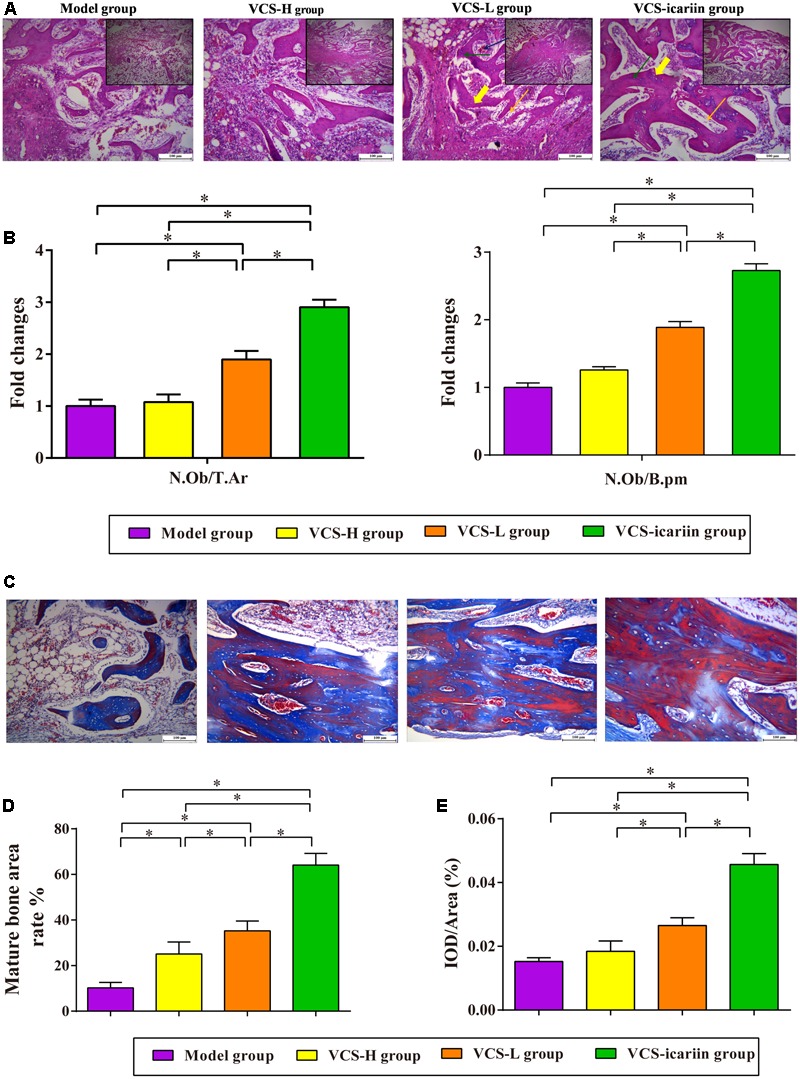
**(A)** Typical histopathology of the rabbit tibia. Yellow bold arrow: bone trabeculae; orange arrow: osteoblasts; green arrow: osteocytes; blue arrow: vessels. **(B)** The columns represent the mean ± SE from five rabbits per group, ^∗^*P* < 0.05. N.Ob: osteoblast number, T.Ar: trabecular bone area, B.pm: bone perimeter. **(C)** Masson’s trichrome staining of the tibia at 8 weeks after treatment with VCS-L, VCS-H, and VCS-icariin. **(D)** The columns represent the mean ± SE of the rate of new bone formation from the analysis of Masson’s trichrome staining. **(E)** Immunohistochemical staining for OC at 8 weeks after treatment with VCS-L, VCS-H, and VCS-icariin. The columns represent the mean ± SD from six rabbits per group, ^∗^*P* < 0.05.

Areas in green, red, and yellow in the fluoroscope images (**Figure 3**) represented regions of calcium precipitation of tissue mineralization labeled by fluorochromes at 7, 14, and 21 days before euthanasia; the deposits of the dyes were seen around the bone defect in the sequence of their administration. As shown in **Table 3**, the MAR and BFR in VCS-icariin group were more than twofold higher than in the VCS-H group during the 21 days before euthanasia. The stimulating effect of icariin on new bone formation was indicated via the increased speed of new bone formation and the quality of new bone. Morphometric analysis indicated a pronounced decrease in N.Ob/T.Ar and N.Ob/B.pm induced by *S. aureus*, but this decrease was mitigated by VCS-L, VCS-H, and VCS-icariin treatment. Moreover, N.Ob/T.Ar and N.Ob/B.pm in the VCS-icariin group were more than twofold higher than in the VCS-L and VCS-H groups. N.Ob/T.Ar and N.Ob/B.pm were significantly higher in the VCS-L group than in the VCS-H group (**Figures 4A,B**). Masson’s Trichrome staining confirmed that the defect in the model group was filled with fibrous tissue. In the VCS-L, VCS-H and VCS-icariin groups, newly formed osteoid tissue and woven bone were easily identified, and the interface between the nascent bone and the host bone was still distinguishable (**Figure 4C**). The mature bone area rates were 10.25 ± 2.36% in the model group, which were significantly lower than in the three treatment groups. The mature bone area rates in the VCS-icariin group was sixfold higher than the model group, and threefold higher than the VCS-H group. The Masson’s Trichrome staining results revealed more mature bone tissue in the VCS-icariin group and less mature bone tissue in the VCS-L and VCS-H groups (**Figure 4D**). Both H&E and Masson’s Trichrome staining results revealed a decrease in the number of osteoblasts and the amount of mature bone tissue in the VCS-L and VCS-H groups. In the VCS-icariin group, there were more osteoblasts and mature bone tissues around bone trabeculae compared with the VCS-H group.

**Table 3:** The dynamic parameters of the tibia with triple fluorescent labeling (*n* = 3).

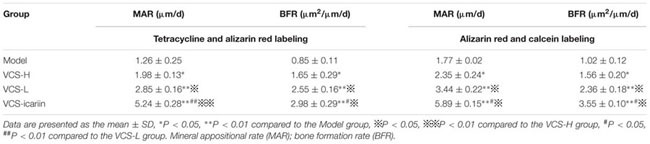

The immunohistochemical analysis of OC in the tibia tissue indicated weaker positive staining for OC in the model group, which can be attributed to *S. aureus* infection (*P* < 0.05). There was no significant difference between the VCS-H group and the model group, indicating that bone repair was inhibited in the VCS-H group. The level of positive staining for OC in the tibia samples in the VCS-icariin group was 0.46 ± 0.003%, significantly higher than in the VCS-L group. In addition, there was a significant difference between the VCS-L group and the VCS-H group (**Figure 4E**).

### Icariin Reversed the Inhibition of ROBs Caused by Vancomycin

**FIGURE 5:**
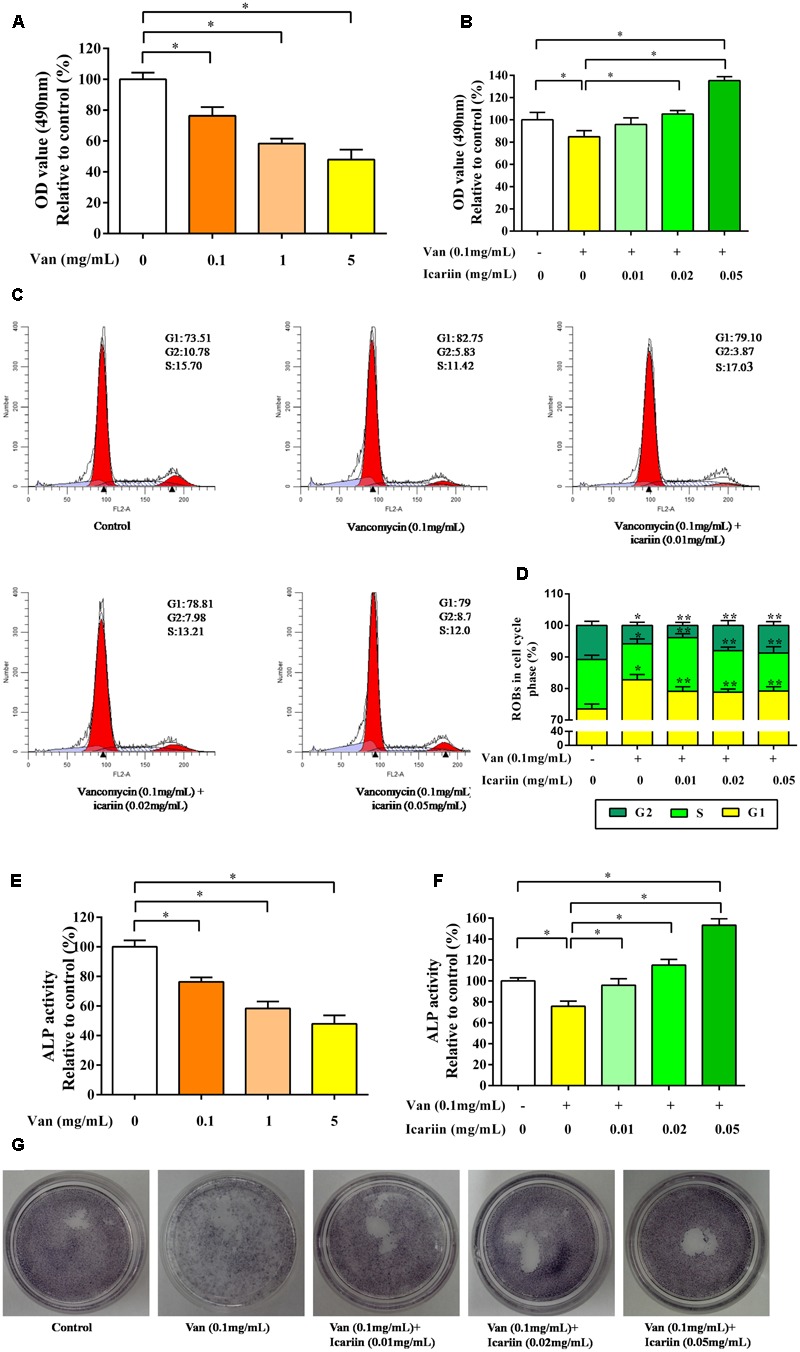
**(A)** The proliferation capability of ROBs treated with vancomycin, ^∗^*P* < 0.05. **(B)** ROBs treated with vancomycin and icariin, ^∗^*P* < 0.05. **(C)** Cell cycle analysis was performed by flow cytometry using propidium iodide DNA staining of ROBs. **(D)** The percentages of ROBs in the cell cycle phases were shown in the percentage histogram. Each value represented mean ± SD of three independent experiments. ^∗^*P* < 0.05, compared to the control ROBs, ^∗∗^*P* < 0.05 compared to ROBs treated with vancomycin. **(E)** ALP level of ROBs treated with vancomycin, ^∗^*P* < 0.05. **(F)** ALP level of ROBs treated with vancomycin and icariin, ^∗^*P* < 0.05. **(G)** ALP positive staining of ROBs treated with vancomycin and icariin.

As shown in **Figure 5A**, ROBs treated with vancomycin at 0.1, 1, and 5 mg/mL exhibited considerable changes in cell morphology and cell proliferation. The proliferation capability of ROBs treated with 0.1 mg/mL vancomycin was reduced to approximate 80% compared to the control ROBs. Icariin significantly increased the proliferation capability of ROBs induced with 0.1 mg/mL vancomycin (**Figure 5B**). As evident from the results (**Figures 5C,D**), 0.1 mg/mL vancomycin increased the percentage of ROBs in the G1 phase of the cell cycle and decreased the percentage of cells in G2 and S phases. However, treatment with 0.01, 0.02, and 0.05 mg/mL icariin led to increase cell cycle arrest in the G2, S phases, meanwhile, G1 phase was reduced significantly, as compared to the ROBs treated with 0.1 mg/mL vancomycin.

Alkaline phosphatase activities in ROBs induced with vancomycin were significantly reduced compared to the control ROBs (**Figure 5E**). Nevertheless, icariin dramatically increased ALP activity at doses of 0.01, 0.02, and 0.05 mg/mL (**Figure 5F**). ALP positive staining also showed that icariin increased ALP activity in osteoblasts, compared to the group without icariin (**Figure 5G**).

Taken together, vancomycin could reduce cell proliferation and differentiation; when its concentration exceeded 0.1 mg/mL, icariin could reverse the inhibition of ROBs.

### Reversal Mechanism of Icariin on Osteoblasts Inhibited by Vancomycin

**FIGURE 6:**
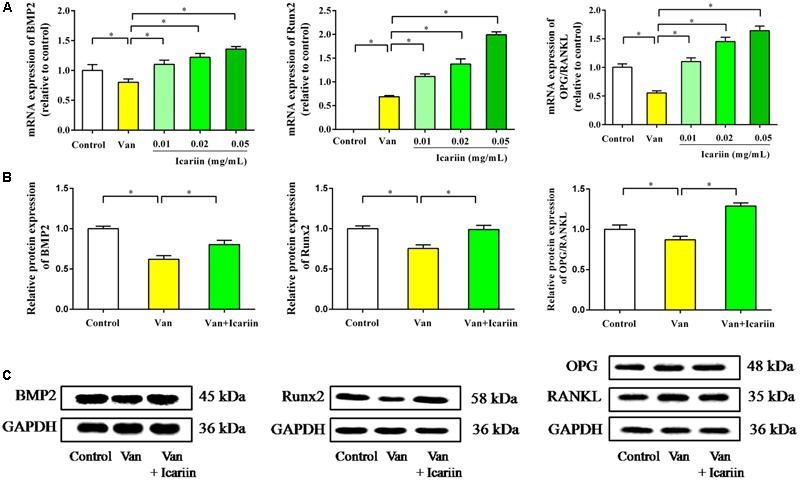
**(A)** RT-PCR results, ^∗^*P* < 0.05. **(B)** Western blot analysis, ^∗^*P* < 0.05. **(C)** Protein expression in ROBs after co-culture with vancomycin and icariin.

RT-PCR data in **Figure 6A** showed that vancomycin at doses of 0.1 mg/mL significantly reduced mRNA expression of BMP2 and Runx2 in ROBs, which demonstrated the inhibitory effect of vancomycin on ROBs. The ROBs co-cultured with vancomycin and icariin significantly enhanced the mRNA expression of BMP2 and Runx2 in ROBs, compared with ROBs co-cultured with vancomycin. As shown in **Figures 6B,C**, the western blot of BMP2 and Runx2 proteins revealed the same results as gene expression. Western blot data showed that the treatment of ROBs with vancomycin at 0.1 mg/mL and icariin at 0.05 mg/mL significantly enhanced the protein expression of BMP2 and Runx2, compared with ROBs that were treated with vancomycin.

RT-PCR results showed that ROBs treated with vancomycin had a lower ratio of OPG/RANKL, and icariin at doses of 0.01, 0.02, and 0.05 mg/mL showed an appreciable effect on the OPG/RANKL ratio. The western blot results showed that the ROBs treated with icariin had a higher level of OPG protein expression and a lower level of RANKL protein expression than the ROBs treated with vancomycin, which produced a higher OPG/RANKL ratio in the ROBs treated with icariin. In summary, the results described above demonstrated that icariin treatment can reverse the inhibitory effect of a high dose of vancomycin on ROBs by regulating the dynamic balance of bone resorption and bone formation via the activation of BMP signaling and the inhibition of the combination of RANKL and RANK.

## Discussion

Vancomycin is the most effective antibiotic for the treatment of bone infection with *S. aureus*. However, many reports indicated that high concentration of vancomycin inhibited bone healing and decreased the number of osteoblasts. Approximately 10–15% of patients with bone infection experienced a long bone union process, delayed union or non-union after anti-infection treatment via the local placement of VCS. In our previous studies, the local concentration of vancomycin in patients with single 100 mg dose could reach a very high level, probably exceed the inhibitory concentration of vancomycin on osteoblasts. In the clinic, a higher dose of VCS was always implanted into the local site, which induced a long bone union process, delayed union or non-union after anti-infection treatment in patients. The current knowledge of Chinese medical plants suggests that they are a viable source of potential osteoprotective agents. For example, *Herba Epimedii* exerted osteoplastic effects on bone union and was identified as a potential treatment for delayed union and non-union of the bone. In previous studies, the main component in *Herba Epimed*ii, flavonoids such as icariin, epimedins A, B, and C were shown to be the major contributor to osteoplastic effects on osteoporosis, osteoarthritis and other bone disease (Zhou et al., 2013). Icariin was shown to be the most effective component, which produced the most significant promoting effect on the proliferation in osteoblasts, and activated estrogen receptor alpha (ER) and stimulate ER-dependent osteoblastic functions (Meng et al., 2005; Jin et al., 2013; Xiao et al., 2014). We preliminarily tested the osteoplastic effect of icariin at different doses (5.43, 10.86, and 21.72 mg/kg/day), found that 10.86 mg/kg/day was the effective dose. In this study, the mean serum concentration-time curves showed that icariin was absorbed into the blood and maintained at an effective concentration of 50 μg/L. Based on the above findings, we hypothesized that high doses of vancomycin inhibited bone healing at the local site, and icariin exerted potential osteoplastic effects on osteoblasts, which could provide a new therapeutic method for delayed union or non-union after anti-infection treatment via the local placement of VCS in bone infection patients.

To examine the hypothesis in this study, The rabbits induced with *S. aureus* were treated with VCS-L, VCS-H and VCS-icariin. The Micro-CT data demonstrated that the shortest symphysis time was in the VCS-icariin group among the three treatment groups, and a shorter symphysis time was detected in the VCS-L group compared to the VCS-H group. Morphometric analysis results indicated that the number of active osteoblasts lining the bone trabeculae and the areas of mature bone tissues were improved after icariin treatment, although there were fewer osteoblasts and less mature bone tissue in the VCS-H group than in the VCS-L group. The triple fluorescence labeling test showed that the speed of bone formation in VCS-icariin group was higher than in the VCS group and was lower in the VCS-H group than in the VCS-L group. All of these results suggested the inhibition of bone healing by a high concentration of vancomycin, and the activation of bone remodeling with icariin. Icariin elevates new bone formation capability and accelerates the speed of bone mineralization around the bone defect locally. Since OC was secreted by osteoblasts and accumulated in the extracellular matrix of bone, it was considered one of the sensitive markers associated with bone formation. In this study, at the end of treatment with VCS-L and VCS-H, and VCS-icariin, the VCS-H group exhibited less positive staining for OC, and the VCS-icariin group exhibited more positive staining for OC than other groups.

*In vitro*, the results showed that the proliferation of ROBs was inhibited by blocking the cell in the G1 phase, when cells were co-cultured with different concentrations of vancomycin, and a positive correlation between this effect and vancomycin concentration was apparent. However, icariin treatment led to increase cell cycle arrest in G2/S phase with prolonged incubation time. We concluded that icariin can affect the proliferation of osteoblasts by promoting DNA synthesis. During early stages of osteoblast differentiation, osteoblasts synthesize ALP and other osteoblastic differentiation markers, ultimately leading to the induction of extracellular matrix calcification (Babey et al., 2015). In this study, the ALP activity was inhibited by treated with 0.1 mg/mL vancomycin, and effectively elevated by icariin during osteoblast differentiation. Moreover, a positive correlation between this effect and the icariin concentration was observed. However, in previous studies, cell numbers were reduced by 50% compared with the control group at 24 h after they were co-cultured with 0.4 mg/mL vancomycin and human dural fibroblast (Goldschmidt et al., 2016). The differences might be due to the variety of species and genera of the cells, and the different characteristics of cellular metabolic activity. High concentrations of vancomycin inhibited mitochondrial energy via the regulation of lactate in the glycolysis process, and different cell lines showed various metabolic characteristics (Duewelhenke et al., 2007; Philp et al., 2017). Although the inhibitory concentrations of vancomycin were diverse in previous studies, the dose dependent tendency of inhibition on the proliferation of osteoblasts was obvious. In addition, cell proliferation and differentiation were investigated at 24 h after co-culture with vancomycin and ROBs in this study, and the quantity and metabolic function of ROBs in the following period of time deserve further research.

**FIGURE 7:**
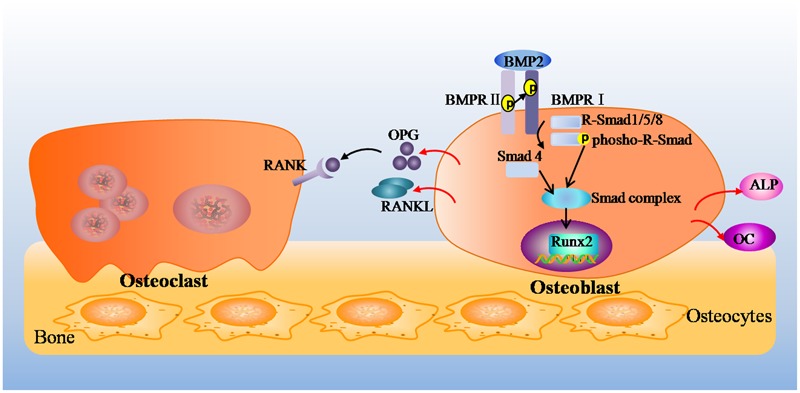
The mechanism of OPG/RANKL pathway and BMP2/Runx2 pathway on bone repair.

Bone remodeling is a cellular process mediated by osteoclasts and osteoblasts, and this process normally occurs in a tightly regulated sequence of events, where the amount of formed bone equals the amount of resorbed bone, thereby completely restoring the removed bone (Chen et al., 2015). Osteoclasts differentiate from monocyte/macrophage lineage precursors under regulation of two cytokines, macrophage-colony-stimulating factor and RANKL (Xu et al., 2016). RANKL binds to its receptor RANK, which promotes the differentiation, multinucleation, activation, and survival of osteoclasts. OPG, a soluble decoy receptor for RANKL, inhibits osteoclastogenesis by blocking the RANKL–RANK interaction, which inhibits osteoclast formation and differentiation and induces apoptosis (Delnaz et al., 2010; Liò et al., 2012). Furthermore, OPG and RANKL are fundamental proteins secreted by mature osteoblasts (Szentpétery et al., 2017). Base on the above, any modification in the OPG-to-RANKL ratio can induce either excessive bone resorption or, in contrast, excessive bone formation. In short, Osteoblasts and osteoblastic lineage cells modulate the formation and activity of osteoclasts by expressing RANKL and OPG, which induce and inhibit osteoclastogenesis, respectively (Krum et al., 2010). On the other hand, BMPs, belong to the transforming growth factor β (TGF-β) superfamily, which have been identified as inducers of ectopic bone and cartilage formation (Yang et al., 2014). BMP2 is one of the most important cytokines playing important roles in bone development and fracture repair (Jang et al., 2012). As shown in **Figure 7**, BMP2 binds to the cell surface type I receptor (BMPR I), type II receptor (BMPR II) to form a complex, then the complex phosphorylatate receptor-activated Smad (R-Smad 1/5/8). The activated Smads combine with Smad 4 (Co-Smad), which come into the nucleus, start-up Runx2 and Osterix expression, up-regulate a variety of special osteoblast mRNAs and protein expression, such as ALP, collagen I, OC, osteopontin, finally promote the synthesis of bone matrix and bone formation (Matsubara et al., 2008; Chen et al., 2012). In brief, BMP2 deems to control the expression and functions of Runx2 through Smad signaling and be essential for osteoblast differentiation (Derek et al., 2015).

The results of the mRNA and protein expression showed that the gene expression of BMP2 and Runx2 and the ratio of OPG/RANKL were decreased in the ROBs by different concentrations of vancomycin and were increased in ROBs by icariin treatment. Based on the results presented above, it was hypothesized that vancomycin inhibited the proliferation and differentiation of ROBs by inhibiting the BMP2/Runx2 pathway and OPG/RANKL pathways, which were the dominant factors that regulated bone formation and bone resorption. Meanwhile, icariin stimulated this balance by up-regulating BMP2 and Runx2 expression. Icariin up-regulated OPG and down-regulated RANKL mRNA and protein expression, leading to an increased OPG/RANKL ratio. Whether the balance between bone formation and bone resorption fully mediated the effects of icariin *in vivo* merits to be further investigated.

## Conclusion

We demonstrated that the local implantation of VCS can control infection in model rabbits; however, a high local concentration of vancomycin significantly reduced bone formation. Icariin exhibited osteoplastic properties on osteoblasts that had been inhibited with high doses of vancomycin *in vitro*, thus indicating that icariin may be worthy of further investigation as a potential agent for the treatment of delayed union and non-union of the bone after anti-infection treatment with vancomycin in bone infection patients.

## Author Contributions

YZ and LS wrote the manuscript. YZ, ZM, NW, XW, and XH performed the experiments, YH, DS, and CW conceived or designed the studies. All authors contributed to analyzing the data.

## Conflict of Interest Statement

The authors declare that the research was conducted in the absence of any commercial or financial relationships that could be construed as a potential conflict of interest.

## Abbreviations

ALP: alkaline phosphatase
BFR: bone-formation rate
BMD: bone mineral density
BMP: bone morphogenetic protein
B.pm: bone perimeter
BV/TV: ratio of bone volume/tissue volume
CRP: high sensitive C-reactive protein
ELISA: enzyme linked immunosorbent assay
H&E: hematoxylin and eosin
MAR: mineral appositional rate
MTT: methyl thiazolyl tetrazolium
N.Ob: osteoblast number
OC: osteocalcin
OPG: osteoprotegrin
PI: propidium iodide
RANKL: nuclear factor-kB ligand
ROBs: rat calvarial osteoblasts
ROI: regions of interest
Runx2: runt-related transcription factor 2
*S. aureus*: *Staphylococcus aureus*
T.Ar: trabecular bone area
VCS: vancomycin-calcium sulfate
VCS-H: high dose VCS
VCS-L: low dose VCS
WBC: white blood count
